# Impact of Anatomical Site on RNA-Based Molecular Subtypes in Paired High-Grade Serous Ovarian Carcinoma Samples

**DOI:** 10.3390/cancers18132115

**Published:** 2026-06-30

**Authors:** Karolin Heinze, Tia S. Murdoch, Evan Cairns, Derek S. Chiu, Aline Talhouk, Ulrich Canzler, Jalid Sehouli, Sven Mahner, Philipp Harter, Jacobus Pfisterer, Stefan Kommoss, Michael S. Anglesio

**Affiliations:** 1 Department of Obstetrics and Gynecology, University of British Columbia, Vancouver, BC V6T 1Z4, Canada; 2 Department of Gynecology and Obstetrics, Medical Faculty and University Hospital Carl Gustav Carus, Technische Universität Dresden, 01307 Dresden, Germany; 3 National Center for Tumor Diseases (NCT) Dresden, 01307 Dresden, Germany; 4 Arbeitsgemeinschaft Gynäkologische Onkologie e.V. (AGO), 82024 Taufkirchen, Germany; 5 Department of Gynecology with Center of Oncological Surgery, Charité, Medical University of Berlin, 13353 Berlin, Germany; 6 Department of Obstetrics and Gynecology, University Hospital, Ludwig-Maximilians-University Munich, 80377 Munich, Germany; 7 Department of Gynecology and Gynecologic Oncology, Evang. Kliniken Essen-Mitte, 45136 Essen, Germany; 8 Center for Gynecologic Oncology, 24103 Kiel, Germany; 9 Department of Obstetrics and Gynecology, Diak Klinikum, 74523 Schwäbisch Hall, Germany

**Keywords:** HGSOC, PrOTYPE, metastasis, microenvironment, molecular subtype

## Abstract

**Background**: High-grade serous ovarian carcinoma (HGSOC) can be subdivided into four prognostic molecular subtypes based on gene expression: C1/Mesenchymal (C1.MES), C2/Immunoreactive (C2.IMM), C4/Differentiated (C4.DIF) and C5/Proliferative (C5.PRO), each representing distinct biological characteristics with immune and stromal microenvironments. PrOTYPE enables prognosis and treatment guidance from biopsy material. Metastatic biopsies are often more accessible than primary adnexal sampling; their utility assumes stable tumor-intrinsic properties relative to the primary. Metastases may diverge due to microenvironmental pressure as well as the site-specific subtype dynamics. **Methods**: Treatment-naïve HGSOC specimens from 138 patients were profiled using the 55-gene nanostring PrOTYPE assay at adnexal, contralateral adnexal, and/or metastatic sites. **Results**: Adnexal PrOTYPE yielded expected distributions (21% C1.MES, 31% C2.IMM, 23% C4.DIF, 25% C5.PRO) with moderate reproducibility (κ = 0.49). Same-site replicate analysis showed substantial reproducibility (κ = 0.7). Non-adnexal sites were enriched for immune/mesenchymal subtypes (C1.MES/C2.IMM, 36/63 cases), most prominently at the omentum (24/32 C1.MES). C5.PRO was distinctly underrepresented at non-adnexal sites. Subtype shifts from adnexal to extra-adnexal sites were enriched for the second-place adnexal type prediction (*p* < 0.001). Detailed 55-gene analysis showed *POSTN/CTSK* were most commonly upregulated across metastatic sites. EMT pathway enrichment increased with metastatic distance (from adnexa to omentum, adj *p* < 0.05), paralleling—but independent of—C1.MES predominance. **Conclusions**: Adnexal PrOTYPE showed good stability. However, non-random subtype shifts and EMT enrichment at metastatic sites suggest dissemination selects pre-existing transcriptional plasticity rather than acquiring states de novo as HGSOC adapts to new microenvironments. Microenvironment changes may help predict metastatic potential and should be considered for precision medicine targeting.

## Simple Summary

High-grade serous ovarian cancer often spreads to distant sites like the omentum, making these metastatic tumors easier to biopsy than the original ovarian tumor. We show that the stratification tool PrOTYPE, used to classify tumors and guide treatment, gives different results depending on which body site is sampled. In general, tumors at the primary site (ovary/adnexa) showed a diversity of gene expression subtypes that remained stable on re-sampling. In contrast, metastatic sites frequently shifted toward more aggressive mesenchymal signatures driven by local tissue environments. Our findings reveal that tumor molecular signatures are highly influenced by anatomical location, highlighting the tumor microenvironment’s selective pressure during progression and metastasis.

## 1. Introduction

Ovarian cancer is the sixth leading cause of cancer-related death [1]. In 2023, 338,615 women worldwide were newly diagnosed with ovarian cancer, and an estimated 65% of these women will ultimately die from their disease, making ovarian cancer the deadliest gynecologic malignancy, with 220,979 deaths in the same period [1,2]. Ovarian carcinomas are well recognized to represent five major and distinct diseases named predominantly based on their histopathological appearance. Amongst the five histotypes, high-grade serous tubo-ovarian carcinoma (HGSOC) accounts for both the majority of cases (~70%) as well as the highest mortality [3,4,5]. At a molecular level, it is defined by a near-universal mutation in *TP53*, deficiencies in homologous repair pathways (with notable somatic and inherited dysfunction/mutation in *BRCA1/BRCA2* genes), genomic instability with high levels of DNA copy number change and chromosomal rearrangements, and aggressive coelomic metastasis [6].

HGSOC can be further divided into subtypes based on additional molecular characteristics. One of the more well-known molecular subtyping schemes was first described by Tothill and colleagues in 2008, using high-density gene expression arrays and applying unsupervised clustering to a large clinical sample cohort of ovarian carcinomas [7]. Validation and refinement of this protocol by TCGA and others established a four-subtype framework where microenvironment and cancer intrinsic features contribute to underlying patterns of gene expression and subsequently validate underlying prognostic value [6,8,9,10]. However, early techniques required a large cohort of samples in order to assign molecular subtypes using clustering and bench methods that were amenable to fresh/frozen research samples rather than standard archival tissues, more common in hospital pathology labs. These drawbacks were overcome in 2020 when Talhouk et al. developed and validated PrOTYPE, a reduced, gene-expression-based classifier derived from subtype-specific gene signatures. PrOTYPE assigns each sample to one of the established molecular subtypes based on the relative expression of a minimal gene set, enabling robust classification from routine clinical material, including formalin-fixed paraffin-embedded (FFPE) tissues. This framework facilitates the application of molecular subtyping in translational and clinical contexts [11].

The consensus subtypes defined by PrOTYPE now recognize C1.Mesenchymal (C1.MES) as having the worst patient outcomes, associated with reactive desmoplasia, metastasis and miliary patterns, which makes surgical intervention difficult. Tumors of C2.Immunoreactive (C2.IMM) and C4.Differentiated (C4.DIF) subtypes demonstrate more favorable outcomes, with both having signatures related to immune function. C2.IMM, in particular, shows heavy infiltration of CD8+ lymphocytes while C4.DIF expresses a greater number of biomarkers associated with epithelial differentiation. C5.Proliferative (C5.PRO) has outcomes only marginally superior to C1.MES, is almost entirely devoid of immune signatures, and instead expresses proliferative and oncofetal markers including *HMGA2*, *MYCN* and the microRNA regulator LIN28B [7,8,9,10,11].

Additional investigations of molecular subtype included evaluating the subtype of a primary tumor (taken from the adnexa) and a metastatic tumor taken from the omentum. It was found that between the adnexal site, which had a fairly even distribution of the four subtypes (range 18–33%), and the omentum, there was a significant shift in subtype, with 72% of cases being classified as C1.MES at the omentum [11]. While the majority of gene expression subtyping studies have not monitored the sampling site for primary HGSOC, this imbalance was noted in a sub-analysis from the original Tothill et al. study [7]. It has become clear that HGSOC modifies the host peritoneal microenvironment as it disseminates [12,13]. However, it is unclear if gene expression subtype predictions remain stable at different metastatic sites or if the desmoplasia commonly observed at omental metastasis is more widespread. This is of particular clinical interest given the prognostic value of PrOTYPE subtypes; the underlying molecular features of subtypes may be useful to guide precision medicine, and the shift to neoadjuvant chemotherapy over primary debulking surgery.

This paper aims to evaluate how molecular subtypes shift between adnexal and other extra-adnexal metastatic sites and how consequent different sampling sites could influence the molecular subtype predictions and utility.

## 2. Materials and Methods

### 2.1. Patient Cohort

Treatment-naïve tissue samples were selected from the OVCARE gynecological tissue bank, Vancouver, Canada and the AGO-OVAR17 trial [14]. Patients provided informed consent and complied with the Declaration of Helsinki; all research was conducted in accordance with the Canadian Tri-Council Policy Statement on Ethical Conduct of Research Involving Humans (TCPS2, 2022). Inclusion criteria required the availability of a HGSOC research biospecimen from both the adnexa and an extra-adnexal site.

A total of 323 specimens from 150 cases were initially selected for examination. Histology review during processing resulted in relabeling 5 cases (10 specimens) as LGSOC and subsequent exclusion. The final cohort analysis was conducted on 279 unique specimens originating from 138 patients (Supplementary Figure S1). In addition, technical and/or biological replicates (as noted in the text) were available for 28 samples from 26 cases, and 7 of these were incomplete cases, accounting for the difference between the initially selected material and the final unique analytical cohort.

### 2.2. RNA Extraction and NanoString

RNA was extracted using the modified Qiagen miRNeasy FFPE kit (Qiagen, Hilden, Germany) as described previously [15]. Quantification of RNA concentration was performed by Nanodrop (Thermo Scientific, Wilmington, DE, USA). Gene expression profiles were assessed using the nCounter analysis system (NanoString, Seattle, WA, USA) according to previously published protocols [11,15]: input of 500 ng total RNA with A260/280 readings between 1.85 and 1.95.

### 2.3. Subtype Classification

PrOTYPE Web Tool [11] (https://ovcare.shinyapps.io/PrOType/ (accessed last on 22 July 2025)) was used to quality control sample output and apply the molecular subtype prediction modeling on the raw count data set retrieved from the nCounter, retrieving the subtype prediction, normalized counts, and entropy scores. Concordance between primary and secondary tumor sites was assessed for each patient. In the event that a unique specimen was run on the NanoString more than once (*n* = 26), we continued with the readout that showed the lowest entropy for downstream analysis.

### 2.4. Statistical Analysis

Statistical analysis of the effects of the PrOTYPE prediction, as well as the individual gene expression counts, was performed in R. Paired differential expression analysis was performed using limma (v3.64.3). Cohen’s kappa and related agreement statistics were calculated using irr (v0.84.1). Data manipulation and visualization were performed using standard packages such as dplyr, tidyr, ggplot2, and ggalluvial (v0.12.6). Variance partitioning was performed to quantify the relative contributions of patient, site, and residual variation to gene expression profiles, using a linear modeling framework integrated with limma-based preprocessing. Gene set enrichment analysis was performed with fgsea (v1.34.2) using Hallmark gene sets from MSigDB (v25.1.1). Differential expression between subtypes or other groupings was ranked using t-statistics generated by limma, and no fold-change threshold was applied prior to pathway analysis. Differential expression between subtypes or other groupings was ranked using moderated t-statistics generated by limma, and no fold-change threshold was applied prior to pathway analysis. Pathway enrichment was evaluated across the complete ranked gene list. Statistical significance for differential expression and pathway enrichment was assessed using FDR-adjusted *p*-values (adj *p*) calculated using the Benjamini–Hochberg method. Statistical significance was defined by *p* < 0.05. Exact *p*-values are reported where appropriate; values below 0.001 are presented as *p* < 0.001.

## 3. Results

### 3.1. Cohort Characteristics

A total of 279 tumor specimens from 138 cases were classified as HGSOC and passed quality control, with complete paired expression data across primary and secondary sites for the PrOTYPE assay of 50 prediction genes and five housekeeping genes (the latter excluded after QC and normalization) [11]. The majority of cases had only two sample specimens available for evaluation—135/138 cases. Amongst all analyzed samples (*n* = 279), the molecular subtype proportions were as follows: 31.9% C1.MES, 29.7% C2.IMM, 19% C4.DIF, 19.4% C5.PRO. These differed from our previous large-scale distribution, particularly for C2.IMM and C4.DIF (χ^2^ = 16.7, *p* < 0.001; Table 1A, Supplementary Table S1). Likewise, the adnexal distribution varied from our previous report, with slightly lower proportions of C1.MES and C4.DIF but elevated C2.IMM and C5.PRO (χ^2^ = 12.2, *p* = 0.0069; Supplementary Table S1).

### 3.2. Anatomical Site-Specific PrOTYPE Observation

Anatomic sites were collapsed to common regions for comparison: ovary (*n* = 138), adnexa (*n* = 58), fallopian tube (*n* = 15) became adnexa; parametrium (*n* = 4), uterus (*n* = 2), serosa (*n* = 6) became uterus; spleen (*n* = 1), pelvis restriction (*n* = 3), peritoneum (*n* = 14), umbilicus (*n* = 1) became peritoneum; all other anatomical site retained their pathological report site annotation resulting in omentum (*n* = 32) and lymph node (*n* = 1) annotations (Table 1A). PCA and UMAP analyses demonstrated clustering primarily by anatomical site, with secondary separation according to PrOTYPE subtypes, suggesting both strong site-specific expression patterns and subtype-specific signals (Supplementary Figure S2).

Amongst the cases that originated from any adnexal site, 20.9% were C1.MES (*n* = 44), 31.3% C2.IMM (*n* = 66), 23.2% C4.DIF (*n* = 49) and 24.6% as C5.PRO (*n* = 52) (Table 1A,B). Non-adnexal sites were heavily enriched for C1.MES (64%), while a few samples were classified as C4.DIF (6.25%) or C5.PRO (3.13%). Samples from the omentum site heavily influenced that observation, as half of the non-adnexal samples (32/64) were omental, and the majority (24/41) were C1.MES classified (Table 1A).

### 3.3. Reproducibility of PrOTYPE Subtype Predictions

**Figure 1:**
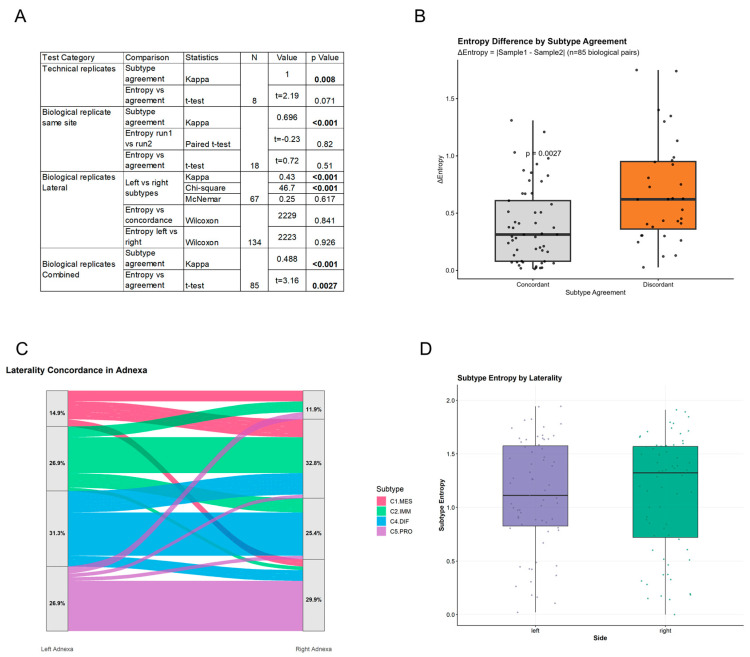
PrOTYPE reproducibility across technical and biological replicate classes. (**A**) Summary of subtype agreement, kappa statistics, and entropy-based comparisons across technical, biological same-site, contralateral, and combined biological replicate classes. Technical replicates showed perfect agreement, whereas biological replicates showed progressively lower agreement, with contralateral pairs contributing most strongly to the combined biological reproducibility estimate. (**B**) Box plots of entropy differences (ΔEntropy) stratified by concordant (agreement) versus discordant (disagreement) subtype calls in combined biological replicates showed a significant difference (*p* = 0.0027). (**C**) Left–right adnexal subtype concordance is shown as an alluvial plot, where the left side represents the left adnexa and the right side represents the right adnexa. Ribbon widths reflect the distribution of subtype calls between sites. No statistical difference between the sides was seen (χ^2^ = 0.25, *p* = 0.617, with 58.2% agreement of subtypes). (**D**) Subtype entropy by laterality in adnexal samples. Entropy was similar between left and right adnexa, consistent with the lack of a significant laterality effect (*p* = 0.926).

PrOTYPE demonstrated excellent technical reproducibility, based on duplicate runs of the same extraction samples (*n* = 8 pairs, 100% agreement, κ = 1.0, *p* = 0.008; Figure 1A). Biological reproducibility was assessed across 85 pairs comprising samples that originated from independent extractions from the same tissue type. This included re-extractions from the same tissue block (adnexal and non-adnexal samples, *n* = 18) and samples with left–right adnexal laterality (*n* = 67). Considered together, the overall biological reproducibility was moderate (Figure 1A, 62.4%, κ = 0.49, *p* < 0.001), whereby concordant pairs had significantly lower entropy differences than discordant pairs (t = 3.16, *p* = 0.0027; 0.394 vs. 0.688, Figure 1B).

Examining contralateral adnexal samples alone, 39/67 (58.2%) showed subtype concordance between right and left adnexa (Figure 1C, Table 1C). Agreement was non-random but only modest (κ = 0.43, *p* < 0.001). The paired subtype table showed a strong left-right association (χ^2^ = 46.70, *p* < 0.001), while the symmetry test did not support a large side-specific shift (*p* = 0.617). Left- and right-side subtype distributions were broadly similar (*p* = 0.76), though C4.DIF appeared more frequently on the left (31.3% vs. 25.4%) and C2.IMM on the right (32.8% vs. 26.9%; Figure 1C). Entropy differences were significant across contralateral pairs, with discordant pairs having higher entropy (*p* = 0.0054; Supplementary Figure S3B though entropy distribution was not different by laterality (W = 2223, *p* = 0.926, Figure 1D).

The reproducibility of independent extraction (same site) biological replicates was considerably higher than contralateral pairs, exceeding 77.8% (κ = 0.696, *p* < 0.0001) and consistent with previous reports [11]. The entropy difference between replicate assays did not differ significantly between concordant and discordant pairs within these same-site biological replicates (*p* = 0.42; Supplementary Figure S3A).

### 3.4. Subtype Concordance and Anatomical Shifts

**Figure 2:**
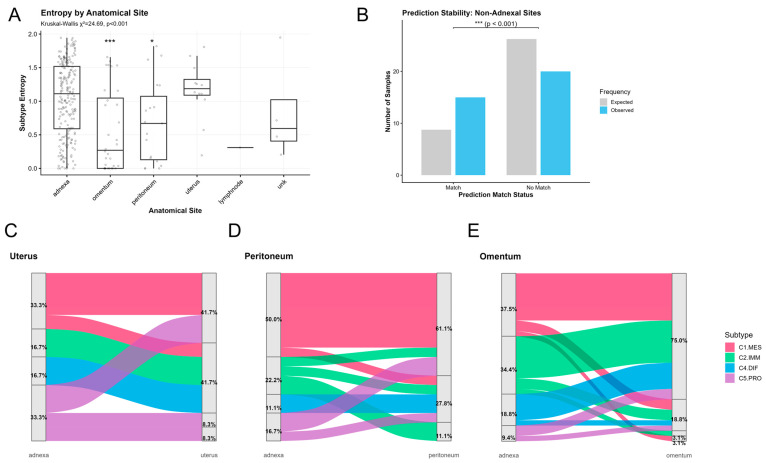
Site-specific PrOTYPE subtype dynamics and prediction stability across anatomical sites. (**A**) Subtype entropy by anatomical site. Entropy distribution was greatest, and with the highest mean entropy at the adnexal site, indicating generally higher heterogeneity and lower prediction confidence at the primary site. Entropy was significantly lower at metastatic sites, particularly the omentum (Kruskal–Wallis *p* < 0.001; omentum vs. adnexa *p* < 0.001). (**B**) Prediction stability in non-adnexal and adnexal samples, comparing observed “match” to the second-highest predicted subtype from the adnexal specimen, being the predicted subtype at the non-adnexal site (no-match = different from subtypes predicted). Observed vs. expected frequencies assumed a random distribution of 25% per subtype, corresponding to equal chance assignment among the four PrOTYPE subtypes. Matches to the second-highest scoring adnexal prediction exceeded expected frequency at non-adnexal sites, while no-match cases were reduced (*p* < 0.001). (**C**–**E**) Alluvial plots showing subtype transitions between anatomical sites. The left side represents the adnexa, and the right side represents the corresponding non-adnexal site: uterus (**C**), peritoneum (**D**), and omentum (**E**). Ribbon widths reflect the proportion of samples assigned to each PrOTYPE subtype at the primary and secondary site, and ribbon colors indicate subtype identity. Kappa values are summarized in Supplementary Table S2. Percentages within each node indicate the distribution of subtypes at that site. Across metastatic sites C1.MES was enriched, whereas C5.PRO was depleted. ***: *p* < 0.001; *: *p* < 0.05.

Building on the C1.MES enrichment observed at the omentum (75.0%; Table 1A), subtype distributions differed significantly across anatomical sites (χ^2^ = 61.36, *p* < 0.001). Entropy also varied strongly by site (Kruskal–Wallis χ^2^ = 24.69, *p* < 0.001, Figure 2A), driven by higher adnexal entropy compared to omentum and peritoneum (Wilcoxon *p* < 0.001) and a marginal difference versus uterus (*p* = 0.078). Subtype concordance varied at extra-adnexal sites: comparing adnexa to omentum revealed 34.4% concordance (Table 1B; Figure 2C–E), predominantly reflecting shifts from C4.DIF/C5.PRO to C1.MES (71.4% of discordant pairs). Uterine and peritoneal samples both showed 50% discordance relative to their adnexal counterparts, with most subtype shifts favoring C2.IMM over others (50% and 44% respectively). Taken together, the alluvial plots showed that adnexal C1.MES tumors were the most stable across metastatic events, while C4.DIF and C5.PRO lesions more frequently underwent subtype switching at metastatic sites (Figure 2C–E).

Although some paired samples were discordant by top-call subtype, the secondary site often corresponded to the adnexal sample’s second-highest predicted subtype, indicating that discordance frequently reflected a predictable subtype shift rather than complete loss of correspondence—as would be expected under a random 25% distribution. This pattern is held for non-adnexal secondary sites (*p* < 0.001; Figure 2B).

### 3.5. Individual Gene Expression Analysis

**Figure 3:**
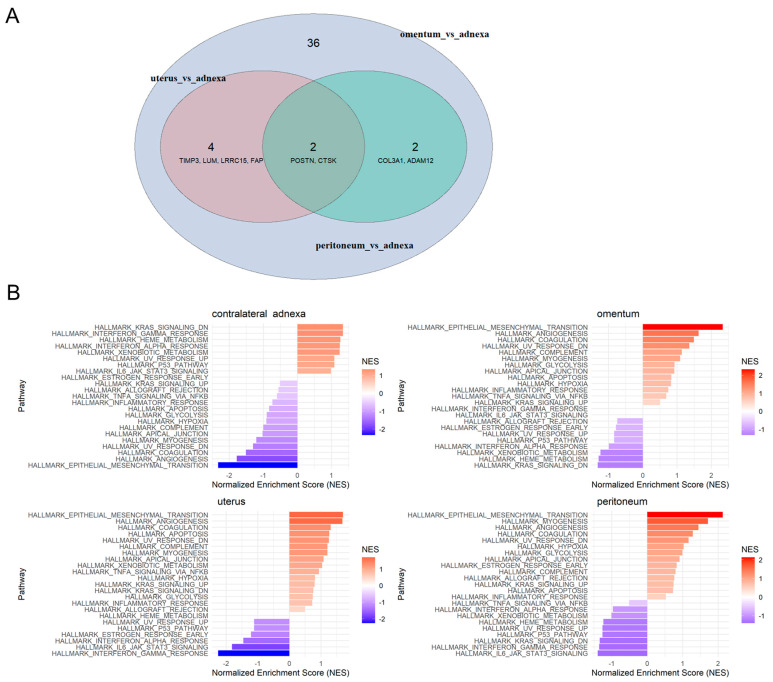
Venn diagram of DEGs and NES plots for GSEA across metastatic sites and adnexal tissue. (**A**) Venn diagram depicting the overlap of DEGs between adnexal tissue (primary reference) and metastatic sites (omentum: 44/55 DEGs; uterus: 6/55 DEGs; peritoneum: 4/55 DEGs). DEGs were defined by adj *p* ≤ 0.05 (Supplementary Table S3). Of these, 36 genes were unique to the adnexa–omentum comparison, with 4 additional genes shared exclusively with the uterus, 2 more shared exclusively with the peritoneum, and 2 further genes (*POSTN*, *CTSK*) common across all metastatic site-adnexa comparisons. DEGs were defined by fold-change ≥ 2 and adj *p* ≤ 0.05 (Supplementary Table S3). (**B**) Composite of GSEA-derived NES plots for gene sets enriched in metastatic samples from four anatomical sites (secondary adnexa, uterus, omentum, peritoneum) relative to primary adnexa. Gene sets were tested using all 55 genes in the NanoString assay, not limited to the significant DEGs. The epithelial–mesenchymal transition pathway was a key enriched feature at omentum (NES 2.3, adj *p* = 0.005), uterus (NES 1.7, adj *p* = 0.014) and peritoneum (NES 2.1 adj *p* = 0.002) contrasting with lack of in contralateral adnexa (NES −2.2, adj *p* = 0.002) and underscoring EMT as a shared metastatic mechanism across sites (FDR q < 0.25, |NES| > 1).

Differential expression analysis using limma was performed to compare gene expression profiles across anatomical sites, modeling a two-level group factor (adnexa vs. non-adnexa), with patient-level blocking using duplicate correlation to account for the paired design (same patient, multiple sites). Pairwise site contrasts were then specified (adnexa vs. omentum, adnexa vs. uterus, adnexa vs. peritoneum). Between the adnexa and omentum, 44 out of 55 tested genes were differentially expressed (adj *p* < 0.05). Among these, *POSTN* and *CTSK* were also differentially expressed in both the adnexa–uterus and adnexa–peritoneum comparisons. An additional six genes from the adnexa–omentum set (*COL3A1*, *ADAM12*, *TIMP3*, *LUM*, *LRRC15*, and *FAP*) overlapped with DEGs from either the adnexa–uterus or adnexa–peritoneum contrasts (Figure 3A). All eight overlapping genes were consistently upregulated at non-adnexal sites (positive log fold-change, adj *p* < 0.05; Figure 3A, Supplementary Table S3). Variance partitioning with per-gene mixed-effects models indicated that, on average, 38% of expression variance (range 15.3–66.8%) was attributable to patient, 7.5% (0–20%) to anatomical site, and the remaining >50% (27.7–68.8%) to residual variation (Supplementary Table S4).

Consistent with the site-associated expression patterns above, entropy derived from PrOTYPE subtype probabilities was strongly correlated with gene expression across the 50 informative genes (Supplementary Figure S4A). Among these genes, significant entropy-associated features showed negative Spearman correlations, consistent with higher expression in lower-ambiguity samples (Supplementary Figure S4B). The strongest entropy-associated genes were predominantly ECM-related (Supplementary Figure S4C).

### 3.6. Non-Adnexal Sites Show Consistent Pathway Reprogramming

Despite the small gene set, gene set enrichment analysis (GSEA) using Hallmark MSigDB gene sets and ranked lists from limma t-statistics (10,000 permutations) identified site-specific pathway enrichments (Figure 3B). Epithelial–mesenchymal transition (EMT) was consistently enriched in non-adnexal sites relative to adnexa (omentum Normalized Enrichment Score (NES) 2.33, adj *p* = 0.005; peritoneum NES 2.13, adj *p* = 0.002; uterus NES 1.70, adj *p* = 0.014). Angiogenesis and coagulation were not statistically significant but in trend enriched in omentum (NES 1.63, adj *p* = 0.21; NES 1.49, adj *p* = 0.41) and peritoneum (NES 1.45, adj *p* = 0.30; NES 1.28, adj *p* = 0.43). Immune pathways such as interferon-γ response were depleted in non-adnexal sites (negative NES), while *IL6/JAK/STAT3* signaling showed site-specific depletion (uterus NES −1.82, adj *p* = 0.011). EMT was absent in contralateral adnexa samples (NES −2.2, adj *p* = 0.002).

## 4. Discussion

HGSOC is well known for intrinsic tumor heterogeneity [16]. The work presented here suggests this extends to interaction with the tumor microenvironment (TME) and gene expression subtype classifications. Our group and others have suggested that TME is altered as HGSOC metastasizes with coelomic spread throughout the peritoneum and omentum [11,17,18,19,20]. This spread and other distant metastasis are more often than not the driver of morbidity for this disease.

Herein, we have confirmed PrOTYPE is a stable gene expression classifier, with excellent technical and biological reproducibility when performing assessment from the same biological site. Amongst samples showing discrepancies between biological replicates at the same site, we noted significantly greater change in entropy in the prediction (compared to biological replicates with consistent subtyping, 0.84 ± 0.23 vs. 0.78 ± 0.25), indicating borderline predictions rather than random technical error. Notably, 75% of discordant pairs (3/4) showed entropy >0.75 (see also Supplementary Figure S3), confirming that prediction instability specifically affects uncertain cases near subtype decision boundaries.

We have shown that PrOTYPE gene expression subtypes change in a systematic pattern as HGSOC disseminates. In the context of contralateral ovarian/adnexal lesions, we did not have a basis to evaluate the true “primary” source, but we also did not see a significant shift in PrOTYPE dependent on laterality. This aligns with HGSOC’s typical primary presentation pattern: bilateral disease in 66–75% of cases due to early intraperitoneal spread rather than independent primaries. Among unilateral HGSOC, right- and left-sided tumors occur at similar rates (~50/50) [21]. This symmetric bilateral pattern is distinct from other gynecological conditions exhibiting laterality, like, e.g., endometriosis [22], ectopic pregnancy [23], or ovarian torsion [24]. PrOTYPE’s moderate left–right concordance (κ = 0.431) thus reflects reported HGSOC biology: dissemination across adnexa from shared clonal origin, without evidence of anatomical laterality bias.

Systematic shifts in PrOTYPE were more pronounced with greater distance from the adnexa. Omentum and other non-adnexal sites favored C1.MES with almost a complete loss of C5.PRO subtype characteristics. The shift was driven by increased expression of *POSTN* and *CTSK,* though all commonly differentially expressed genes from any pairwise adnexal vs. non-adnexal analysis were either direct ECM components (*COL3A1*), modulators of ECM binding (*ADAM12*, *LUM*, *LRRC15*), or mediators of ECM degradation/deposition (*POSTN*, *CTSK*, *TIMP3*, *FAP*). Most of these genes have also been implicated in the promotion of metastasis and EMT of ovarian carcinomas [25]. These genes largely define the C1.MES signature and unsurprisingly pointed to gene set enrichments around EMT, and angiogenesis around extra-adnexal lesions, as may be expected in the context of aggressive tissue invasion and damage leading to an immune-reactive and desmoplastic tumor environment. The concordant increase in EMT-associated signals from adnexal to non-adnexal sites further supports a site-associated shift toward a more mesenchymal and pro-metastatic state.

PrOTYPE signatures at the adnexa showed both the greatest diversity in subtype distribution and entropy, generally higher entropy than extra-adnexal lesions. Amongst lesions where divergence was observed from adnexa to extra-adnexal lesions, we saw a significant association between the predictor’s adnexal second choice subtype and top-ranked prediction at the extra-adnexal site, suggesting the ranking predictions at the adnexal site may hint at metastatic potential. This observation raises the possibility that subtype probability distributions and secondary subtype rankings may contain additional biological information beyond the top-ranked subtype assignment alone. If validated, these measures could potentially serve as indicators of transcriptional plasticity and identify tumors with increased propensity for site-associated subtype transitions or metastatic behavior. From a translational perspective, this may support future risk stratification approaches that incorporate prediction confidence or subtype mixtures rather than discrete subtype classification alone. However, these findings should be interpreted cautiously, given the cross-sectional design and targeted gene panel used in this study. Future validation may include integration with spatial or single-cell transcriptomic approaches, assessment of longitudinal samplings, or pre-/post-treatment sampling strategies to determine whether subtype rankings across time or anatomical site are clinically informative.

Our data are consistent with the concept that PrOTYPE and the underlying gene expression subtypes first described by Tothill et al., TCGA, and others [6,7,8,9,10] reflect TME interactions rather than purely tumor cell-intrinsic expression programs, suggesting that an intrinsically aggressive clone may already be present in the primary site and subsequently selected or amplified in the metastatic niche. This proposition implies a tubal/adnexal primary; however, the current study design does not permit inference regarding clonal selection or temporal acquisition of these states. This concept is being expanded in recent work from our group, wherein stromal and epithelial compartments were analyzed separately and show that C1.MES and C2.IMM are entirely dominated by stroma gene expression profiles (manuscript submitted [26]). Similarly, others have shown that HGSOC tumors’ transcriptional plasticity is at least in part influenced by site-specific microenvironmental pressures [27,28,29,30]. In this context, C1.MES clones influence the pro-metastatic/pro-desmoplastic TME, particularly observable at the omentum but consistent across extra-adnexal metastasis. Given the limited gene panel, immune-feature downregulation at metastatic sites was less obvious than in previous reports [31,32,33]. These findings may also have implications for future clinical implementation of transcriptomic classifiers. As neoadjuvant chemotherapy (NACT) and pre-surgical biopsy strategies become increasingly common in HGSOC management [34,35], molecular profiling is frequently performed on limited biopsy material rather than adnexal tumor bulk. Omental biopsies are often favored due to accessibility [36]. Our observations suggest that subtype assignment may vary across anatomical sites and therefore may not fully reflect the transcriptional state of disease elsewhere. Furthermore, treatment-associated transcriptional changes following NACT may alter subtype assignment and complicate interpretation of molecular classifiers in interval debulking settings [36,37,38]. This highlights the importance of considering biopsy origin and treatment timing when interpreting PrOTYPE results. It is unusual for the adnexal mass to make up any portion of residual disease (even microscopic), thus future studies should evaluate whether multi-site or site-aware sampling strategies improve the clinical utility of molecular classification approaches.

Nonetheless, the universal enrichment of *POSTN* and *CTSK* at extra-adnexal sites is consistent with cancer-associated fibroblasts (CAF)-driven matrix remodeling [39,40,41] and EMT progression [42]. Others have pointed to a pro-fibrotic, adipocyte-rich omental niche as promoting metastatic behavior [43], and this likely amplifies selection of an aggressively metastatic clone. However, our observation of the underlying C1.MES signatures in adnexal tumors that switch to this dominant profile at metastatic sites suggest an intrinsic tumor (clone) may already be present in the primary site and can initiate aggressive behavior independent of direct influence from the omental niche.

This study should be considered as a hypothesis-generating pilot adding to a growing body of tumor heterogeneity literature and pointing to a greater need to sample not just primary, but metastatic sites to guide a precision patient management strategy [18,19,44,45,46]. The study is limited by a relatively small sample size, particularly for cases with more than two sampled anatomical sites. In addition, we are constrained by the use of a pre-selected targeted gene expression assay rather than genome-wide profiling, and therefore pathway-level inferences should be interpreted as exploratory and require validation in broader transcriptomic datasets. Increasing accessibility of genome-wide spatial genomic/transcriptomic assays in the research setting would enable a more comprehensive understanding of the evolutionary trajectories of aggressive and metastatic HGSOC clones and their interaction with the TME.

## 5. Conclusions

This study demonstrates that molecular subtype assignment in HGSOC is not uniformly conserved across anatomical sites and that metastatic lesions show systematic shifts in transcriptional profiles relative to adnexal disease. In particular, metastatic sites displayed enrichment of mesenchymal-associated signatures and reduced representation of proliferative states, highlighting spatial heterogeneity in tumor and TME-associated gene expression patterns. These findings suggest that molecular subtype classification may be influenced by biopsy location and support consideration of multi-site sampling in future studies evaluating transcriptomic biomarkers. Given the targeted nature of the assay and the pilot design of this study, these observations should be interpreted as hypothesis-generating and require validation in larger cohorts and with broader transcriptomic approaches. Overall, our results are consistent with the presence of spatial transcriptional plasticity in HGSOC and support further investigation of how site-specific biology may influence molecular classification and clinical translation.

## Figures and Tables

**Table 1:** PrOTYPE molecular subtype assignments and concordance analyses. (**A**) Subtype distribution by anatomical site. (**B**) Adnexa vs. non-adnexa subtype confusion matrix. (**C**) Right–left adnexal concordance matrix. Values are presented as counts with percentage in parentheses (total [%]).

**A**	**All Samples**	**Anatomical Site**					
		**Adnexa**	**Uterus**	**Omentum**	**Peritoneum**	**Lymph Node**	**Unknown**
Total [%]	279	211 [75.6]	12 [4.3]	32 [11.5]	19 [6.8]	1 [0.4]	4 [1.4]
C1.MES	89 [31.9]	44 [20.9]	5 [41.7]	24 [75.0]	12 [63.2]	-	3 [75]
C2.IMM	83 [29.7]	66 [31.3]	5 [41.7]	6 [18.8]	5 [26.3]	1 [100]	-
C4.DIF	53 [19.0]	49 [23.2]	1 [8.3]	1 [3.1]	2 [10.5]	-	-
C5.PRO	54 [19.4]	52 [24.6]	1 [8.3]	1 [3.1]	-	-	1 [25]
**B**		**Non-Adnexa**					
		**C1.MES**	**C2.IMM**	**C4.DIF**	**C5.PRO**		
Adnexa	C1.MES	20	4	0	1		
	C2.IMM	9	6	3	0		
	C4.DIF	5	5	0	0		
	C5.PRO	6	2	1	1		
**C**		**Adnexa Left**					
		**C1.MES**	**C2.IMM**	**C4.DIF**	**C5.PRO**		
Right	C1.MES	3	3	0	2		
	C2.IMM	5	10	6	1		
	C4.DIF	0	4	12	1		
	C5.PRO	2	1	3	14		

## Data Availability

Data is contained within the article, or Supplementary Material, and code is available in a publicly accessible repository (https://github.com/EDGEResearch-CA/anatomical_heterogeneity_PrOTYPE (accessed on 25 June 2026)).

## Supplementary Materials

The following supporting information can be downloaded at: https://www.mdpi.com/article/10.3390/cancers18132115/s1, Figure S1: Principle Component Analysis; Figure S2: PCA of PrOTYPE expression profiles; Figure S3: Box plots of entropy differences by subtype agreement across the biological replicate types; Figure S4: Entropy-associated gene expression patterns and correlations across samples; Table S1: Molecular subtype distribution comparison; Table S2: Cohen’s kappa and pairwise McNemar symmetry tests for paired site classifications considering adnexa-metastatic site comparisons; Table S3: List of Significant differential expressed genes across the different metastatic sites; Table S4: Variance-Partition by gene; Table S5: Normalized gene expression data of NanoString PrOTYPE assay in study cohort.

## Abbreviations

The following abbreviations are used in this manuscript:

HGSOC	High-grade serous Ovarian Carcinoma
LGSOC	Low-grade serous Ovarian Carcinoma
adj *p*	Adjusted *p*-value
C1.MES	C1/Mesenchymal
C2.IMM	C2/Immunoreactive
C4.DIF	C4/Differentiated
C5.PRO	C5/Proliferative
NES	Normalized Enrichment Score
GSEA	Gene set enrichment analysis
PrOTYPE	Predictor of High-grade Serous Ovarian Carcinoma Molecular SubTYPE
TME	Tumor microenvironment
EMT	Epithelia-Mesenchymal transition
NACT	Neoadjuvant Chemotherapy
CAF	Cancer-Associated Fibroblast
