# Supplementary figures and images for: Impact of Anatomical Site on RNA-Based Molecular Subtypes in Paired High-Grade Serous Ovarian Carcinoma Samples
Source: Cancers (Basel). 2026 Jun 30;18(13):2115. doi: 10.3390/cancers18132115 (PMC13359785; doi:10.3390/cancers18132115)

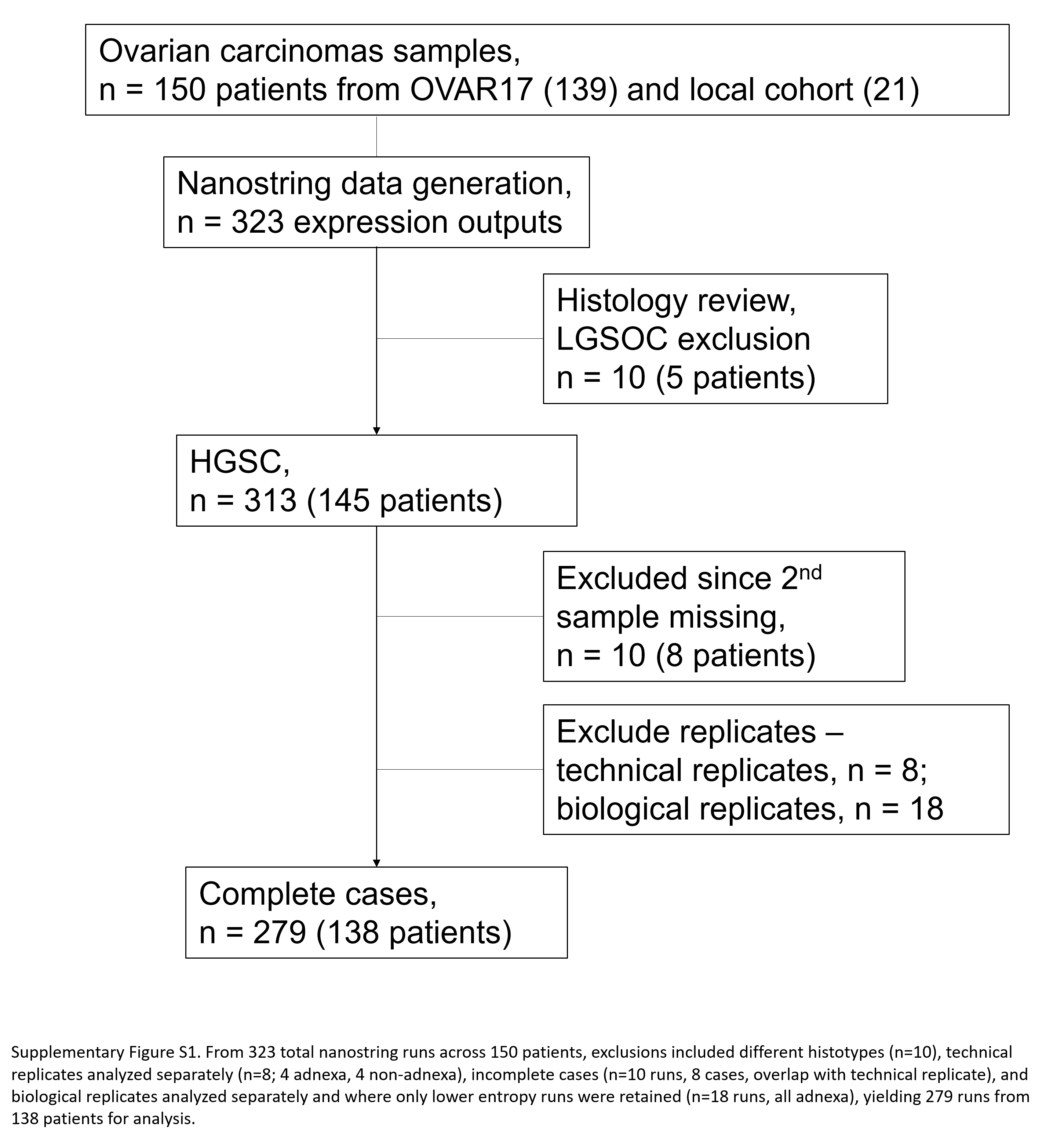

Supplement: Supplementary file 1 [file cancers-18-02115-s001.zip › FigureS1.png]

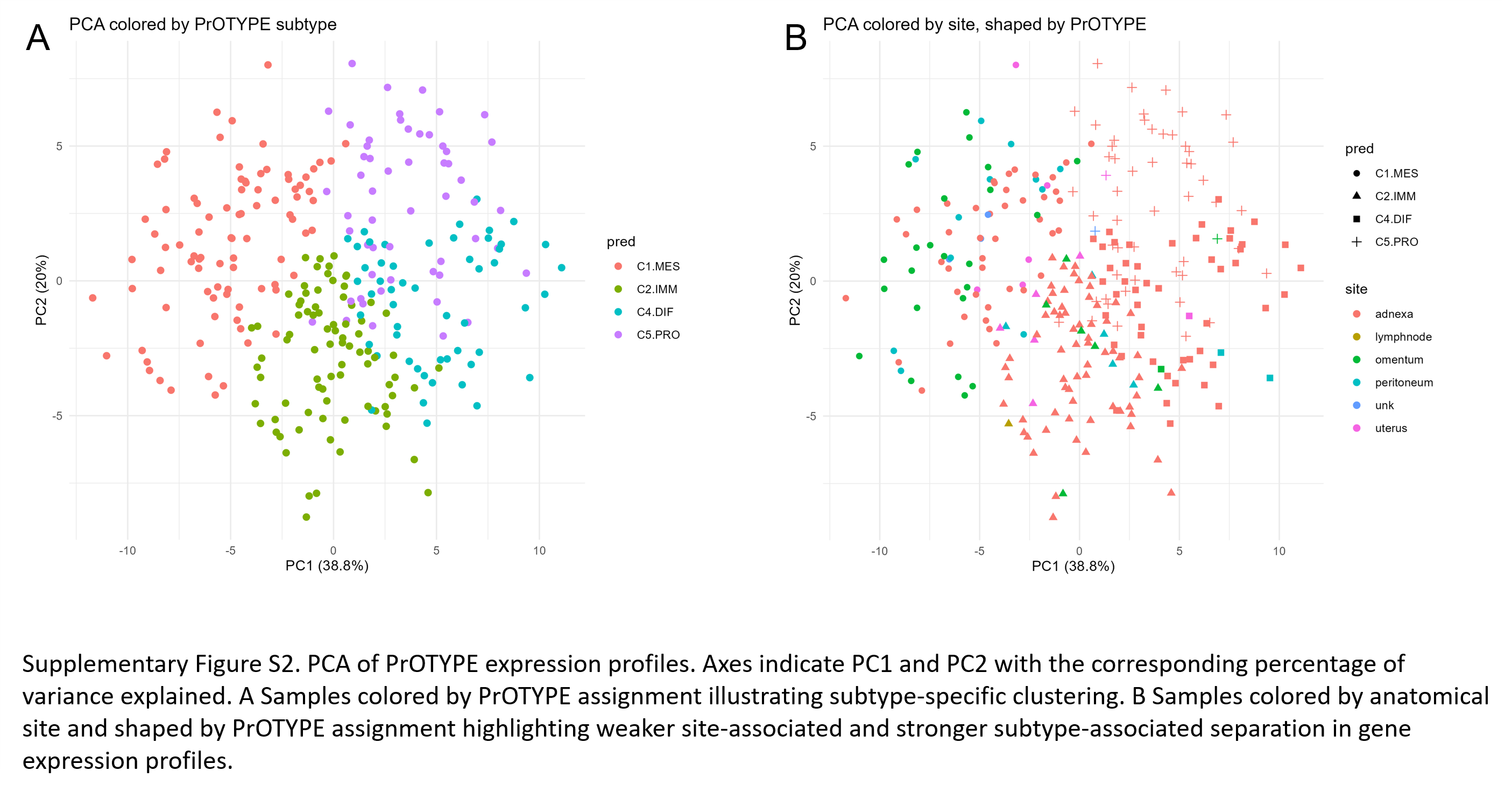

Supplement: Supplementary file 1 [file cancers-18-02115-s001.zip › FigureS2_rev.png]

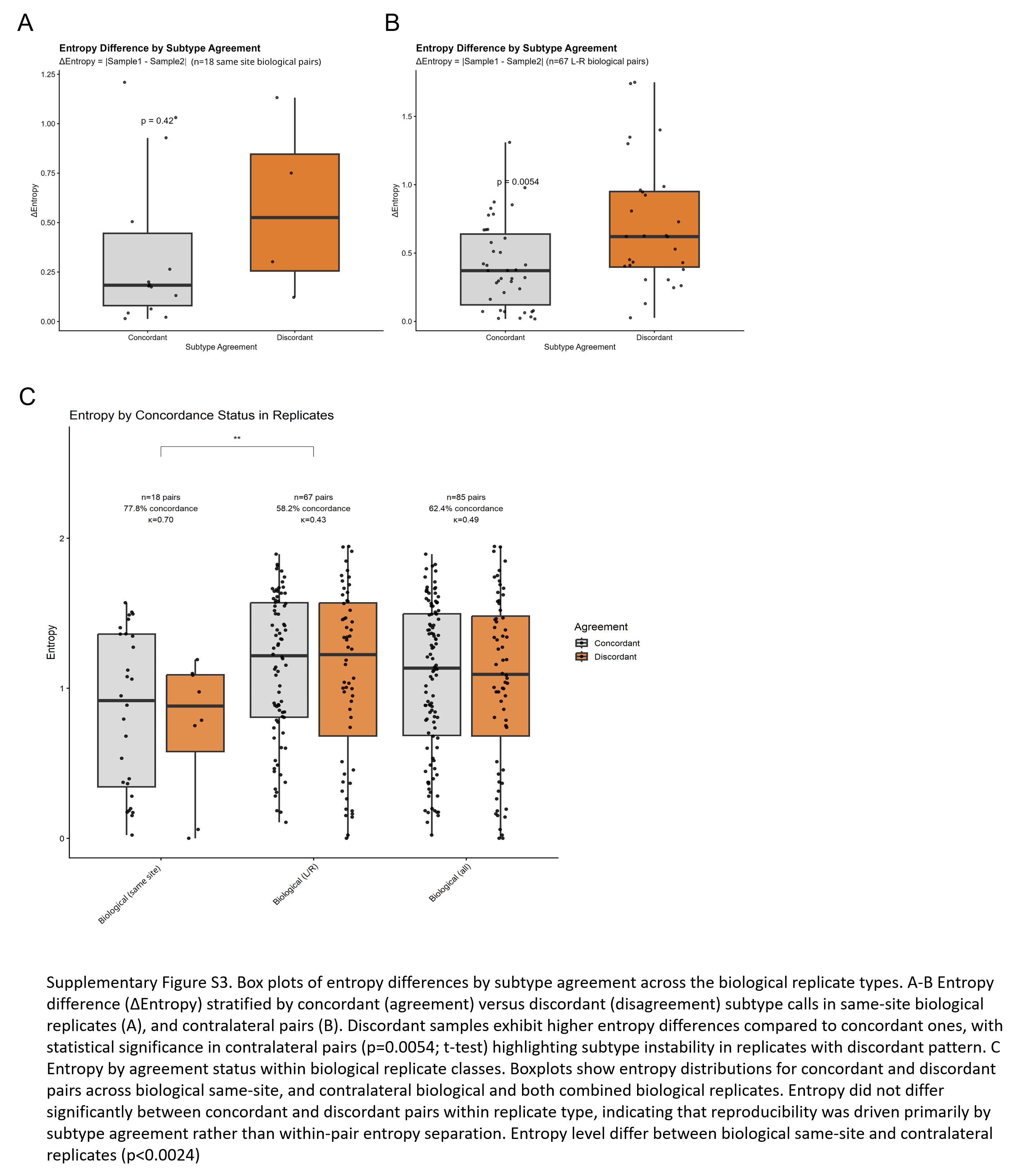

Supplement: Supplementary file 1 [file cancers-18-02115-s001.zip › FigureS3.png]

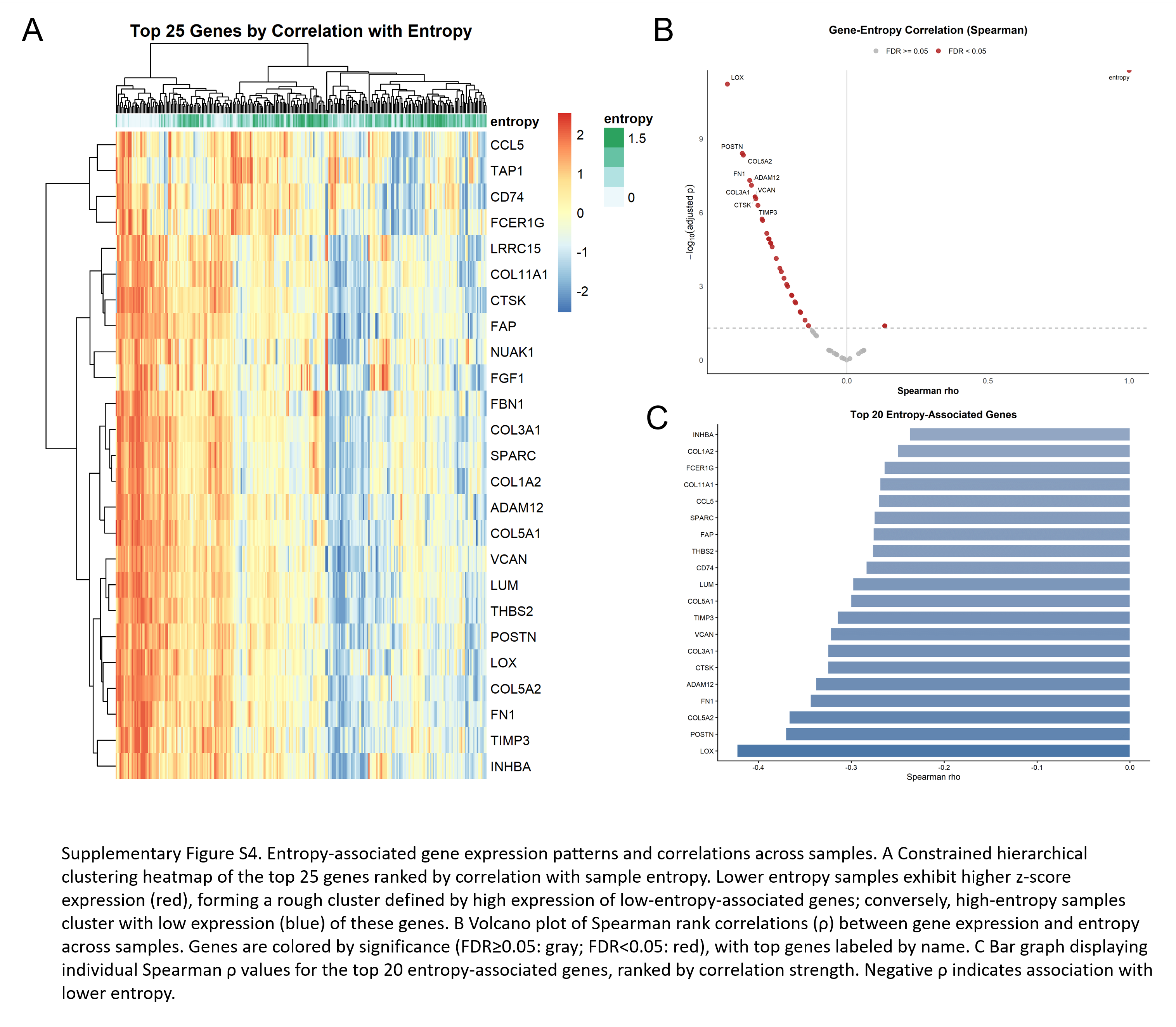

Supplement: Supplementary file 1 [file cancers-18-02115-s001.zip › FigureS4.png]
